# The effect of Ventricular Assist Devices on cerebral autoregulation: A preliminary study

**DOI:** 10.1186/1471-2253-11-4

**Published:** 2011-02-22

**Authors:** Judith Bellapart, Gregory S Chan, Yu-Chieh Tzeng, Philip Ainslie, Adrian G Barnett, Kimble R Dunster, Rob Boots, John F Fraser

**Affiliations:** 1 Department of Intensive Care, Royal Brisbane and Women's Hospital. Butterfield Street, Herston (4029), QLD, Australia; 2 Critical Care Research Group and Department of Intensive Care Medicine, The Prince Charles Hospital and University of Queensland, Rode road, Brisbane, (4032), QLD, Australia; 3 Biomedical Systems Laboratory, School of Electrical Engineering and Telecommunications, University of New South Wales, Sydney, NSW, 2052, Australia; 4 Cardiovascular Systems Laboratory, Department of Surgery and Anesthesia, University of Otago, 23 A Mein Street, Newtown, PO Box 7343, Wellington, New Zealand; 5 Department of Human Kinetics, Faculty of Health and Social Development, University of British Columbia Okanagan, Kelowna, Canada; 6 Institute of Health and Biomedical Innovation & School of Public Health, Queensland University of Technology, 60 Musk Avenue, Brisbane, (4059), Australia; 7 Critical Care Research Group, The Prince Charles Hospital. Medical Engineering Research Facility, Queensland University of Technology, Australia

## Abstract

**Background:**

The insertion of Ventricular Assist Devices is a common strategy for cardiovascular support in patients with refractory cardiogenic shock. This study sought to determine the impact of ventricular assist devices on the dynamic relationship between arterial blood pressure and cerebral blood flow velocity.

**Methods:**

A sample of 5 patients supported with a pulsatile ventricular assist device was compared with 5 control patients. Controls were matched for age, co-morbidities, current diagnosis and cardiac output state, to cases. Beat-to-beat recordings of mean arterial pressure and cerebral blood flow velocity, using transcranial Doppler were obtained. Transfer function analysis was performed on the lowpass filtered pressure and flow signals, to assess gain, phase and coherence of the relationship between mean arterial blood pressure and cerebral blood flow velocity. These parameters were derived from the very low frequency (0.02-0.07 Hz), low frequency (0.07-0.2 Hz) and high frequency (0.2-0.35 Hz).

**Results:**

No significant difference was found in gain and phase values between the two groups, but the low frequency coherence was significantly higher in cases compared with controls (mean ± SD: 0.65 ± 0.16 vs 0.38 ± 0.19, *P *= 0.04). The two cases with highest coherence (~0.8) also had much higher spectral power in mean arterial blood pressure.

**Conclusions:**

Pulsatile ventricular assist devices affect the coherence but not the gain or phase of the cerebral pressure-flow relationship in the low frequency range; thus whether there was any significant disruption of cerebral autoregulation mechanism was not exactly clear. The augmentation of input pressure fluctuations might contribute in part to the higher coherence observed.

## Background

Ventricular assist devices (VAD) are mechanical pumps that replace or augment left and/or right ventricular function in cases of refractory cardiogenic shock. A number of approaches are currently taken related to the indications of these devices: VAD can be used as a bridge to heart transplantation, as a bridge to myocardial recovery leading in some cases to their prolonged use with meaningful survival and improved quality of life [1]. Recently VAD have also begun to be used as a "bridge to destination" that is, they are the final plan for the patient, being used for many years, until the patient succumbs.

Fundamental differences regarding cardiac output and systemic circulation distinguish two main types of VAD: pulsatile and continuous-flow VAD. The main advantages of continuous-flow VAD being the self-contained nature, not requiring a pneumatic driver, longevity, lack of bearing contacting with blood and absence of artificial valves with theoretically smaller thrombogenic surface [2]. However, the effects of non-pulsatile perfusion on end-organ function remain controversial [3-5]. Pulsatile circulation and its effects on systemic vascular resistances have been related to the improvement of microcirculation and endothelial integrity [6,7]; reduction in splanchnic perfusion and reduction of intestinal edema [8]; improvement of the cerebral haemodynamics and cerebrospinal fluid drainage [2] and the maintenance of neuro-endocrine cascades, specifically within the renin-angiotensine system and catecholamine release [5].

**Figure 1:**
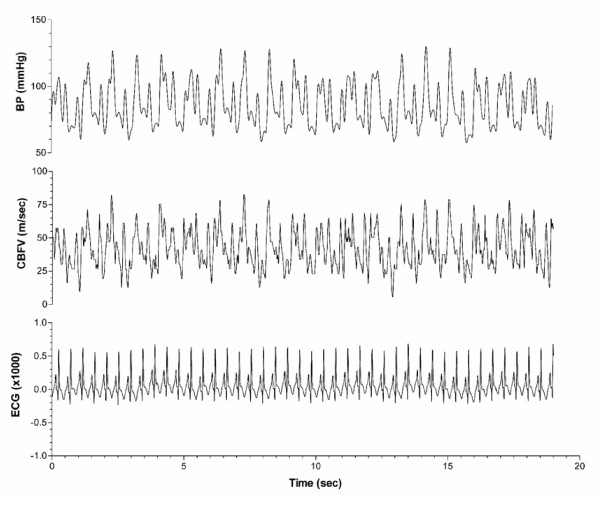
**Real time, beat-to-beat traces of arterial blood pressure (BP) and cerebral blood flow velocity (CBFV) with a ventricular assist device (VAD)**. Upper channel: arterial BP waveform in a patient supported with a VAD, showing irregular fluctuations; middle channel: CBFV (insonated at the level of middle cerebral artery) with fluctuations transmitted from arterial BP; lower channel: electrocardiogram (ECG).

Despite the use of pulsatile VADs, non-homogeneous output is often generated as pulsatile VADs eject once the pre-established filling volume (stroke volume) has been reached. Therefore, the VAD ejection rate varies depending on preload and systemic resistance. Frequently there is a variable degree of persistent native cardiac contractibility, leading to asynchrony, and irregularities in arterial blood pressure waveform (Figure 1). In such situations of circulatory irregularity, end-organ perfusion such as cerebral blood flow may require an intact autoregulation to ensure stable microcirculation.

Cerebral autoregulation is the mechanism by which cerebral blood flow (CBF) is maintained despite changes in cerebral perfusion pressure (CPP). Cerebral autoregulation mediates states of hyperemia and ischemia to avoid vasogenic edema or infarction respectively [9]. Impaired autoregulation has been regarded as a risk factor associated with adverse neurological outcome after cardiac surgery [10,11]. As a dynamic phenomenon, cerebral autoregulation may respond to spontaneous and induced changes in arterial blood pressure (BP) such as those occurring with pulsatile VADs [12,13]. Cerebral autoregulation has been extensively studied using transcranial Doppler (TCD) which measures cerebral blood flow velocities (CBFV) as a surrogate of CBF [14,15] using a variety of methods [16]. From all described methods, transfer function analysis (TFA) enables the analysis of phase shift, gain and coherence between two signals (arterial BP as input and CBFV as output) at a range of frequencies, and has the advantage of being applicable for continuous and non-invasive testing of cerebral autoregulation at the bedside.

Rider and coworkers assessed cerebral autoregulation in patients supported with non-pulsatile VADs, by exposing them to dynamic maneuvers such as head-up tilting and measuring the change in CBFV. They found that cerebral autoregulation was impaired, suggesting that circulatory pulsatility is crucial for the maintenance of cerebral autoregulation [17]. However, their study occurred during the acute phase of the disease, after the insertion of a non-pulsatile VAD and prior to any myocardial "modeling" [18] could have occurred. Some authors have demonstrated that even with the use of non-pulsatile VAD, if a recovery time is allowed, CBF shows recovery of its pulsatility [2], attributing this finding to overall myocardial recovery and specifically right ventricular recovery. Whilst previous study examined the effects of non-pulsatile VAD on the regulation of steady state CBF, this study is the first to investigate the effects of pulsatile VAD, which generates irregular pressure waveform patterns, on the dynamic cerebral pressure-flow relationship by applying the cross-spectral TFA technique.

## Methods

Institutional Ethics Committee approval for the performance of the study was granted. All patients or their next of kin gave informed consent prior to enrolment in the study.

A convenience sample of five patients supported with a pulsatile Thoratec VAD (Thoratec corporation, Pleasenton, CA, US) was compared with five control patients, matched for age, comorbidities, current diagnosis and cardiac output state (Table 1). All cases were supported with a left ventricular pulsatile VAD and inotropic drugs for an average of 7 days. All patients were in their acute phase of their disease. Control subjects were in a low output state requiring inotropic or vasopressor support but without the support of VAD. Although their mean arterial blood pressure (MAP) was similar to the VAD cases, their native left ventricular ejection fraction (LV EF) was better. All patients in the control group survived, whereas 2 of the VAD cases died (Table 2).

**Table 1:** Demographics and patients' characteristics

VAD	Age (years)	Pathology	comorbidities	day of admission
VAD1	52	MI	none	day 2
VAD2	43	MI	hypertension	day 29
VAD3	25	OHCA + MI	none	day 25
VAD4	35	OHCA	hypertension	day 7
VAD5	63	OHCA	hypertension	day 25

Control				

C1	64	MI	none	day 5
C2	65	MI	hypertension	day 4
C3	69	OHCA + MI	hypertension	day 3
C4	55	OHCA	hypertension	day 3
C5	50	OHCA	hypertension	day 2

MI: Myocardial infarct; OHCA: Out of hospital cardiac arrest;

**Table 2:** Therapy and clinical variables

VAD	Support therapy	MAP (mmHg)	CBFV (cm/s)	PCO_2 _(mmHg)	LV EF (%)	Outcome
VAD1	VAD	82	38	36	35	Survived
VAD2	VAD	96	47	40	30	Survived
VAD3	VAD + DPM + NA	60	101	42	20	Intrahospital death
VAD4	VAD	74	45	40	20	Survived
VAD5	VAD + DPM	70	39	32	15	Intrahospital death

Mean ± SD		76 ± 14	54 ± 26	38 ± 4	24 ± 8	

Control						

C1	DPM	79	36	42	40	Survived
C2	DPM	90	43	35	25	Survived
C3	DPM + DBT	70	53	48	30	Survived
C4	DPM + NA	75	39	41	30	Survived
C5	DPM	80	42	37	40	Survived

Mean ± SD		79 ± 7	43 ± 6	41 ± 5	33 ± 7	

*P*		0.74	0.36	0.39	0.09	

VAD: Ventricular Assist Device; MAP: Mean Arterial Pressure; CBFV: cerebral blood flow velocity (mean values are given); LV EF: Left Ventricular Ejection Fraction; NA: Noradrenaline; DPM: Dopamine; DBT: Dobutamine.

We recorded at least 5 minutes of data under resting conditions in all subjects. Simultaneous beat-to-beat recordings of BP and cerebral blood flow velocity (CBFV) waveforms were sampled using a data acquisition unit (ADInstruments, Australia). The BP waveform was acquired from an intra-arterial catheter; CBFV of middle cerebral artery (MCA) was measured using a transcranial Doppler device with a 2 MHz probe and a power of 100 mW/cm^2 ^(DWL, Germany). CBFV of middle cerebral arteries (MCAs) were measured using TCD following referenced criteria at the temporal acoustic window [15]. Both MCAs were insonated and the side with best acoustic characteristics chosen for study. Intra-patient variability was minimized by using only one investigator formally trained in TCD [16]. Stability of the insonated vessel diameter was assumed by maintaining a stable partial pressure of arterial carbon dioxide (pCO_2_) during measurements.

Therapeutic and clinical variables were recorded at the moment of data acquisition. This study was merely observational and did not interfere with the treating physician's management plan.

### Spectral Analysis

For the assessment of cerebral autoregulation, this study used TFA based on frequency domain cross-spectral analysis. TFA assesses the relationship between two signals in the frequency domain and yields three interpretable parameters (i.e. gain, phase, and coherence). Gain is the indicator of the magnitude with which the change of output signal (i.e. CBFV) is caused by the change of input signal (i.e. BP). In the context of cerebral autoregulation analysis, a small gain indicates that cerebral blood flow does not change significantly when blood pressure changes, indicating that the cerebral autoregulatory mechanisms are intact. Phase shift relates to the temporal lag between BP and CBFV at each frequency. Zero phase lag signifies synchronous fluctuations, whilst positive phase suggests CBFV leading BP, and negative phase suggests BP leading CBFV.

The gain and phase metrics, however, need to be interpreted in the context of the cross-spectral coherence, which is an estimation of the linear correlation between the input and output signals at particular frequencies. Coherence varies between 0 and 1; where 0 indicates no linear relationship and 1 indicates perfect linear relationship. It has been suggested that an increase in coherence may be indicative of a blunted cerebral autoregulation [21]. A low coherence, however, can be interpreted as presence of external noise/input, or nonlinear/lack of relationship between input and output.

In this study, spectral analysis was performed on 5 min artifact-free segments of continuous CBFV and BP signals. Signals were downsampled to 1 Hz after appropriate anti-aliasing lowpass filtering, with any slow trend removed by cubic spline detrending. The frequency spectra and transfer function were obtained using the Welch method [21]. This involved subdividing the signal into 120s segments with 75% overlap (resulting in 7 segments), multiplying each segment with a Hanning window, then performing a Fast Fourier Transform (FFT), and finally averaging to give the spectra. Defining the autospectra of BP and CBFV as *S*_xx_(*f*) and *S*_yy_(*f*) (with *f *denoting frequency), the cross-spectrum of BP and CBFV, *S*_xy_(*f*), was computed as the product of *S*_xx_*(*f*) and *S*_yy_(*f*) (*asterisk denotes the complex conjugate*). The transfer function from BP to CBFV was computed as *H*(*f*) = *S*_xy_(*f*)/*S*_xx_(*f*), and the gain magnitude and phase angle of the transfer function was obtained accordingly. The magnitude-squared coherence function was computed as γ^2^(*f*) = |*S*_xy_(*f*)|^2^/*S*_xx_(*f*)*S*_yy_(*f*), for detecting linear correlation between the spectral components in the two signals. Coherence ranged from 0 (*lack of linear correlation*) to 1 (*perfect linear relationship*).

The spectral powers of BP and CBFV and the mean values of the transfer function gain, phase and coherence were calculated in the very low frequency (VLF, 0.02-0.07 Hz), low frequency (LF, 0.07-0.20 Hz) and high frequency (HF, 0.20-0.35 Hz) ranges as previously defined [22]. Unpaired Student's t-test was performed to compare the variables between the VAD and the control groups. *P *< 0.05 was considered statistically significant.

## Results

The patient characteristics are presented in table 1 and 2. No significant difference in MAP, pCO_2 _and LVEF was found between the VAD and the control groups. The levels of pCO_2 _were maintained within normal ranges and stable throughout the study, thus the effect of CO_2 _on cerebral vessel was minimised. LVEF was generally lower for the VAD cases, whichwas expected as these were patients with baseline refractory cardiogenic shock who required a VAD for life support. However the difference did not reached statistical significance.

**Figure 2:**
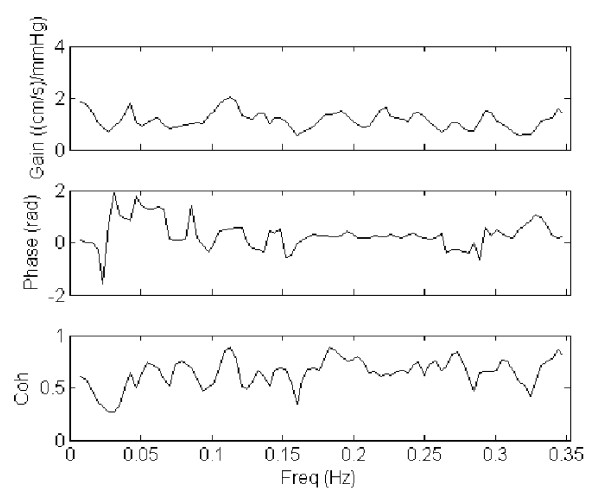
**Transfer function analysis (TFA) of mean arterial pressure (MAP) and cerebral blood flow velocity (CBFV) in a patient with ventricular assist device (VAD)**. The gain, phase and coherence spectra of a representative case with VAD were shown.

**Figure 3:**
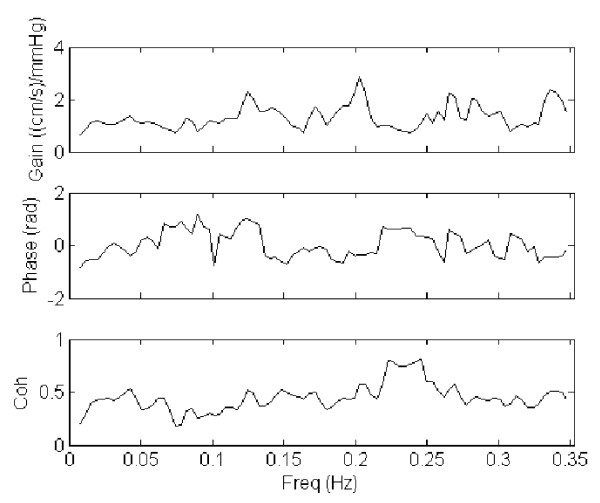
**Transfer function analysis (TFA) of mean arterial pressure (MAP) and cerebral blood flow velocity (CBFV) in a control patient**. The gain, phase and coherence spectra of a representative control were shown.

The results from spectral and cross-spectral transfer function analysis of MAP and CBFV were presented in table 3 and 4. Display of gain, phase and coherence for a representative case and control are shown in figures 2 and 3 respectively. No significant difference was found between the VAD and the control groups, apart from a significantly higher LF coherence between MAP and CBFV in the VAD cases (*P *= 0.04).

**Table 3:** Power spectrum analysis of mean arterial pressure (MAP) and mean cerebral blood flow velocity (CBFV) in ventricular assist device (VAD) cases and controls

VAD	VLF		LF		HF	
	pMAP	pCBFV	pMAP	pCBFV	pMAP	pCBFV
VAD1	0.80	2.77	0.28	0.48	0.94	0.47
VAD2	2.34	7.94	2.41	3.77	0.32	1.46
VAD3	1.06	3.22	0.20	0.94	8.88	3.72
VAD4	3.26	6.55	4.42	3.67	2.15	1.79
VAD5	2.97	2.61	0.49	1.14	5.44	2.24

Mean ± SD	2.09 ± 1.11	4.62 ± 2.46	1.56 ± 1.84	2.00 ± 1.59	3.55 ± 3.58	1.94 ± 1.19

**Control**	**VLF**		**LF**		**HF**	
	**pMAP**	**pCBFV**	**pMAP**	**pCBFV**	**pMAP**	**pCBFV**

C1	0.47	1.12	1.55	0.80	3.80	0.56
C2	1.52	4.92	0.08	0.61	2.44	1.42
C3	2.43	6.93	0.10	0.43	2.55	1.69
C4	0.95	2.72	0.23	0.65	0.17	0.25
C5	0.24	1.20	0.70	1.42	1.98	3.23

Mean ± SD	1.12 ± 0.88	3.38 ± 2.51	0.53 ± 0.62	0.78 ± 0.38	2.19 ± 1.31	1.43 ± 1.17

*P*	0.17	0.45	0.27	0.13	0.45	0.52

VLF, very low frequency (0.02-0.07 Hz); LF, low frequency (0.07-0.2 Hz); HF, high frequency (0.2-0.35 Hz). pMAP (in mmHg^2^) and pCBFV (in (cm/s)^2^) are spectral powers of MAP and mean CBFV respectively.

*P < 0.05 from t-test between VAD and Control.

**Table 4:** Transfer function analysis (TFA) of mean arterial pressure (MAP) and mean cerebral blood flow velocity (CBFV) in ventricular assist device (VAD) cases and controls

VAD	VLF			LF			HF		
	Coh	Gain	Phase	Coh	Gain	Phase	Coh	Gain	Phase
VAD1	0.67	1.35	1.00	0.65	1.20	-0.38	0.67	0.75	0.51
VAD2	0.76	1.82	0.98	0.79	1.25	-0.08	0.68	1.96	0.30
VAD3	0.20	0.95	0.32	0.45	1.52	0.27	0.54	1.13	0.21
VAD4	0.44	0.89	0.90	0.82	0.83	0.51	0.74	0.79	0.16
VAD5	0.45	0.63	1.04	0.56	1.18	0.72	0.77	0.72	0.00

Mean ± SD	0.50 ± 0.22	1.13 ± 0.47	0.85 ± 0.30	0.65 ± 0.16	1.20 ± 0.25	0.21 ± 0.44	0.68 ± 0.09	1.07 ± 0.52	0.24 ± 0.19

**Control**	**VLF**			**LF**			**HF**		
	**Coh**	**Gain**	**Phase**	**Coh**	**Gain**	**Phase**	**Coh**	**Gain**	**Phase**

C1	0.13	0.55	0.73	0.65	0.65	0.62	0.74	0.36	-0.09
C2	0.61	1.43	0.63	0.21	1.71	-0.24	0.32	2.29	0.27
C3	0.72	1.33	0.33	0.30	1.89	0.07	0.34	2.24	-0.15
C4	0.46	1.12	-0.27	0.25	1.07	-0.40	0.28	1.02	-0.18
C5	0.25	1.15	-1.71	0.51	1.18	0.76	0.91	1.28	0.21

Mean ± SD	0.43 ± 0.25	1.12 ± 0.34	-0.06 ± 1.00	0.38 ± 0.19	1.30 ± 0.50	0.16 ± 0.51	0.52 ± 0.29	1.44 ± 0.83	0.01 ± 0.21

*P*	0.65	0.96	0.089	0.039*	0.69	0.88	0.26	0.42	0.11

VLF, very low frequency (0.02-0.07 Hz); LF, low frequency (0.07-0.2 Hz); HF, high frequency (0.2-0.35 Hz). Coh, gain (in cm/s/mmHg) and phase (in rad) are the transfer function coherence, gain and phase from MAP to mean CBFV.

*P < 0.05 from t-test between VAD and Control.

## Discussion

In this study, the cross-spectral transfer function analysis technique was applied to study the dynamic relationship between systemic BP and CBFV in patients using pulsatile VAD. The rationale was to describe any potential alteration of cerebral autoregulation function associated with the use of VAD, as the long term use of VAD may lead to impaired cerebral autoregulation and worse neurological outcomes.

The key finding of the study was the higher coherence between MAP and CBFV in the VAD patients compared with the controls, at the LF range. A low coherence between MAP and CBFV (<0.5)indicates a lack of linear relationship between pressure and flow at the particular frequency range, and can be attributed to the presence of an intact cerebral autoregulation that introduces nonlinearity relationship [21,22]. It has been suggested that the complex nonlinear behavior of the cerebral vasculature might be responsible for the low coherences at the VLF and LF ranges [24-26]. The augmented LF coherence in the VAD patients, on the other hand, might suggest a lower degree of cerebral autoregulation, possibly due to disruption of autoregulatory mechanisms by the use of VAD. However, one potential limitation to this interpretation was that, although no significant difference in the MAP power was observed between the two groups, the two VAD patients with the highest coherence (~0.8) also had much higher spectral power in MAP than the rest of the group. It has been suggested that an increased input pressure change might lead to an increase in coherence, via an improved "signal-to-noise" ratio [27]. This effect might contribute in part to the higher coherence in the VAD group.

The lack of differences in TFA gain and phase between the VAD and the control group also raised questions whether there was significant disruption of cerebral autoregulation by the use of pulsatile VAD. Alterations in cerebral autoregulation function by pathological conditions (such as stroke and autonomic failure [28,29]) are typically associated with changes in gain and/or phase, which were not observed in the current study. Neverthelss, it appeared that the highpass filtering property of the cerebral circulation, characterised by smaller gain at the lower frequencies (VLF) and an increase in gain towards the higher frequencies (HF) [21,27], was more apparent in the control group compared with the VAD group. It would therefore still be possible that gain properties of cerebral autoregulation might have changed in the VAD patients, although the interpretation of the gain parameter would have been limited somewhat by the low coherences in the control patients.

### Methodological considerations and limitations

In this study, direct assessment of CBF was not feasible as the use of non-imaging TCD does not facilitate the measurement of the cerebral vessel cross-sectional area. Instead, there is a global consensus supporting the use of CBFV as a surrogate for CBF, provided the vessel diameter remains stable during the study [30]. Among all factors intervening in changes of vessel diameter and therefore determining CBF [23], pCO_2 _is directly related with vessel diameter and was maintained stable and within normal values, during patient recruitment.

For TCD recordings, only the MCA with better acoustic properties was recorded and analyzed. Although spatial heterogeneity of cerebral perfusion as well as interhemispheric differences has been described [30]; the endpoint in this study was to ensure the best transcranial Doppler recordings in order to minimize the signal-to-noise ratio and increase data reliability [30].

No significant change in gain and phase was found between the two groups in this study, but the small population recruited could have contributed to the lack of statistical significance, thus further studies with larger sample size would be desirable.

## Conclusion

The use of pulsatile VAD affected the coherence but not the gain or phase of the cerebral pressure-flow relationship in the low frequency range, thus whether there was any significant disruption of cerebral autoregulation mechanism was not clear. The augmentation of input pressure fluctuations might contribute in part to the higher coherence observed. Given the absence of all conditions that define autoregulation, these results should be regarded as preliminary data, and further studies, employing bigger samples, are warranted.

## Competing interests

The authors declare that they have no competing interests.

## Authors' contributions

JB and JFF conceived and designed the study; JB undertook patient screen and data acquisition; JB drafted the manuscript which was reviewed and amended by all other authors. GC and Y-C T undertook data analysis and contributed to its interpretation. AGB undertook statistical analysis. KRD conceived and designed the technical components of the study, also reviewed revised the manuscript. RB and PA reviewed and amended the manuscript. All authors read and approved the final manuscript.

## Pre-publication history

The pre-publication history for this paper can be accessed here:

http://www.biomedcentral.com/1471-2253/11/4/prepub
